# A candidate factor that interacts with RF2, a restorer of fertility of Lead rice-type cytoplasmic male sterility in rice

**DOI:** 10.1186/s12284-014-0021-6

**Published:** 2014-10-07

**Authors:** Shinya Fujii, Tomohiko Kazama, Yukihiro Ito, Soichi Kojima, Kinya Toriyama

**Affiliations:** Graduate School of Agricultural Science, Tohoku University, Sendai, 981-8555 Japan

## Abstract

**Background:**

The pollen function of cytoplasmic male sterile (CMS) plants is often recovered by the *Restorer of fertility* (*Rf*) gene encoded by the nuclear genome. An *Rf* gene of Lead rice type CMS, *Rf2*, encodes a small mitochondrial glycine-rich protein. RF2 is expected to function by interacting with other proteins, because RF2 has no motifs except for glycine-rich domain.

**Findings:**

To elucidate the protein that interacts with RF2, we performed yeast two-hybrid screening. We identified four genes and named *RF2-interacting candidate factors* (*RIF1* to *RIF4*). A study of subcellular localization demonstrated that only RIF2 was targeted to mitochondria. A pull-down assay using *E. coli*-produced recombinant GST-tagged RF2 and His-tagged RIF2 confirmed that RF2 interacted with RIF2. *RIF2* encodes ubiquitin domain-containing protein.

**Conclusions:**

These results suggest that RIF2 is a candidate factor of a fertility restoration complex of RF2.

**Electronic supplementary material:**

The online version of this article (doi:10.1186/s12284-014-0021-6) contains supplementary material, which is available to authorized users.

## Findings

Cytoplasmic male sterility (CMS), which is caused by an aberrant mitochondrial gene, is unable to produce functional pollen. Pollen function is often recovered by a *Restorer of fertility* (*Rf*) gene encoded by the nuclear genome. Many *Rf* genes are reported to encode pentatricopeptide repeat (PPR) protein, which is involved in the processing of mitochondrial RNA (Fujii and Toriyama [2008] for a review). Different from PPR-type *Rf* genes, *Rf2,* which is a fertility restorer gene of Lead Rice (LD) type CMS, encodes a mitochondria-targeted 152-amino acid protein containing glycine-rich domain (Itabashi et al. [2011]). *Rf2* is, therefore, considered to restore fertility by a novel mechanism. RF2 is expected to function by interacting with other proteins, because mature RF2 is a small protein that putatively consists of 80 amino acids after removing a predicted mitochondrial targeting signal sequence; it has no motifs except for a 29-amino acid long glycine-rich domain (Itabashi et al. [2011]).

**Figure 1:**
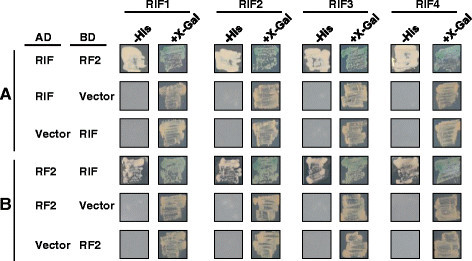
**Interaction between each AD-RIF and BD-RF2 (A), or between AD-RF2 and each BD-RIF (B) by Y2H assay.** Colonies were assayed for histidine autotrophy (-His columns) or beta-galactosidase activity (+X-Gal columns). Vector indicates pGADT7 for AD and pBGKT7 for BD, respectively.

To elucidate a protein that interacts with RF2, we performed Y2H screening. We screened the library (1.8 × 10^6^ clones) made from the mature anther RNA of rice (*Oryza sativa* cv. Taichung 65; Fujii et al. [2009]) using Matchmaker Yeast Two-Hybrid System (Clontech, Tokyo, Japan) according to the manufacturer's protocol. To generate a bait construct, a coding sequence of mature RF2, which lacks a mitochondrial targeting signal sequence, was amplified by PCR using primers Rf2_CDS_EcoRI217F and Rf2_CDS_BamHI456R (primer sequences were shown in Additional file 1: Table S1) and inserted downstream of a sequence for GAL4 DNA-binding domain in pGBKT7. This construct was introduced into yeast strain Y187, and then Y187 was mated with another yeast strain AH109 that contained a prey construct in which cDNA was inserted downstream of a sequence for GAL4 activation domain in pGADT7. Screening was carried out on the SD medium that lacks tryptophan, leucine, histidine and adenine, and is supplemented with 5 mM 3-AT. Positive clones were re-grown on the same SD medium additionally supplemented with X-α-Gal. Interaction was confirmed by re-transformation of yeast. By this screening we obtained 22 positive clones. Nucleotide sequence analysis showed that these clones contained cDNA derived from four distinct genes, which were named *RF2-interacting candidate factors* (*RIF1, RIF2, RIF3* and *RIF4*; Table 1). Interaction between RF2 and RIFs was also confirmed by prey-bait swapping experiments in which RF2 was fused to GAL4-activation domain and each RIF protein was fused to GAL4 DNA-binding domain (Figure 1).

**Table 1:** **Genes identified by Y2H screening**

Name	Number of clones	RAP-DB Locus ID	cDNA accession No.	Annotation
*RIF1*	7	Os04g0404900	AK107531	Conserved hypothetical protein
*RIF2*	2	Os10g0542200	AK065246	Ubiquitin domain containing protein
*RIF3*	10	Os04g0229100	AK071484	Similar to Sinapyl alcohol dehydrogenase
*RIF4*	3	Os12g0507600	AK070613	Conserved hypothetical protein

A fertility restoration factor is expected to function in mitochondria. We therefore investigated the subcellular localization of RIF1, RIF2, RIF3 and RIF4. Coding sequences of RIF proteins were PCR-amplified using primers listed in Additional file 1: Table S1 and using full-length cDNAs obtained from the Rice Genome Resource Center (National Institute of Agrobiological Sciences, Tsukuba, Japan). They were cloned into pENTR D-TOPO vector (Invitrogen, Tokyo, Japan), and then inserted between CaMV 35S promoter and GFP gene in pGWB5 vector (Nakagawa et al. [2007]).

**Figure 2:**
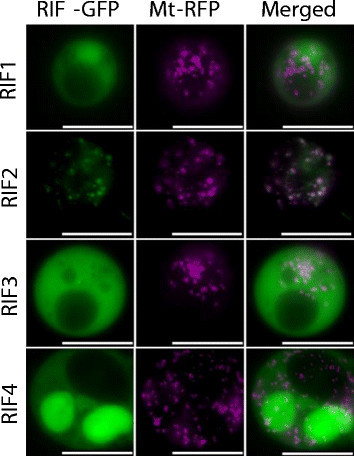
**Subcellular localization of each RIF-GFP.** Subcellular localization of RIF proteins was examined as GFP fusions (RIF-GFP) in rice protoplasts. Mitochondrial-localizing RFP (Mt-RFP) was used as a marker for mitochondrial localization. Scale bars = 10 μm.

Each of the resulting plasmids that express GFP fusion proteins was introduced into rice protoplasts with a plasmid containing CaMV35S promoter-F1F0 ATPase mitochondrial targeting signal-RFP (Arimura and Tsutsumi [2002]) using the polyethylene glycol method (Chen et al. [2010]). GFP fluorescence of RIF2-GFP was co-localized with mitochondrial-localizing RFP, whereas GFP fluorescence of RIF1-GFP, RIF3-GFP and RIF4-GFP was observed in cytoplasm (Figure 2). These results indicated that only RIF2 was targeted to mitochondria.

**Figure 3:**
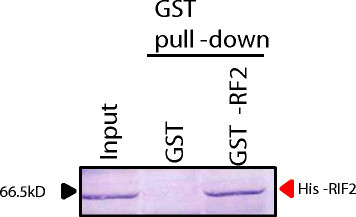
**Pull-down assay to test** ***in vitro*** **interaction between RF2 and RIF2.** Pull-down assay was performed using a His-RIF2 protein with GST (control) or GST-RF2. Input lane contains His-RIF2.

Next we performed pull-down assay using *E. coli*-produced recombinant GST-tagged RF2 (GST-RF2) and His-tagged RIF2 (His-RIF2). The coding sequence of mature RF2 that lacks a predicted mitochondrial targeting signal sequence was PCR-amplified using primers Rf2_CDS_CACC217F and Rf2_CDS_BamHI456R (Additional file 1: Table S1), and inserted into pENTR D-TOPO using Gateway technology (Invitrogen), and then transferred to pDEST17 for a GST fusion protein (Invitrogen). The coding sequence of RIF2 was similarly cloned into pDEST15 for a His fusion protein. Expression of GST-RF2, His-RIF2 and GST alone was induced for 2 h at 37°C by adding L-arabinose. A protein extract of GST-RF2 or GST alone was mixed with a protein extract of His-RIF2, and the GST proteins were purified with Glutathione sepharose 4B (GE Healthcare, Tokyo, Japan). Western blotting using anti-His-tag antibody (Quiagen, Tokyo, Japan) detected His-RIF2 in the purified fraction of GST-RF2, but not in the fraction of GST alone (Figure 3). This result confirms that RF2 interacts with RIF2.

**Figure 4:**
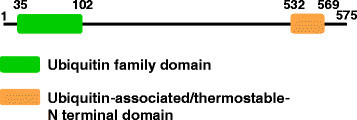
**Motif of RIF2 protein predicted by pfam (** http://pfam.xfam.org/ **) analysis.**

RIF2 encodes a 575 amino acid protein containing ubiquitin family domain and ubiquitin-associated/thermostable-N terminal domain (Figure 4). The most similar protein with known function is ubiqulin 1 (NCBI Protein Accession Nos. NP_444295. 1 for humans and NP_689420. 1 for mice), which is reported to be associated with ubiquitin ligases and proteasomes (Ko et al. [2004]). Although a ubiquitin proteasome proteolysis system has not been reported in mitochondria, we could not rule out the possibility that RIF2 is involved in ubiquitin-proteasome proteolysis of impaired mitochondrial proteins after their translocation to the outer membrane as reported for quality control of human mitochondria (Shanbhag et al. [2012]). In this case, a CMS-causing protein would be a target of degradation for fertility restoration.

Another possibility for RIF2 function in regard to fertility restoration is that RIF2 plays a role in an RNA processing complex. In this case the complex would contain a factor that directly recognizes and processes a target RNA, since neither RF2 nor RIF2 harbors a motif associated with RNA processing. Formation of an RNA processing complex was reported in Hong-Lian CMS. In this CMS, GRP162, which contains a glycine-rich motif and an RNA recognition motif, has been reported to interact with a PPR protein RF5 and unknown components to form a fertility restoration-complex that mediates the processing of CMS-associated *atp6-orf79* RNA (Hu et al. [2012]). RF2 of LD-CMS showed limited amino acid sequence identity (28%) to GRP162 but lacks an RNA recognition motif. The RIF2, which encodes ubiquitin-domain containing protein, might be one of the components of a fertility restoration-complex in the LD-CMS/*Rf2* system. Further study is now in progress to discover other components, which might include RNA recognition/binding proteins in the RF2 fertility restoration-complex that mediate processing of a CMS-associated mitochondrial RNA.

## Authors' contributions

KT and TK conceived and designed the experiments. SF performed the experiments and drafted the manuscript. KT and YI revised the manuscript. YI and SK supervised Y2H experiments. All authors read and approved the final manuscript.

## Additional file

## Electronic supplementary material

Additional file 1: Table S1.: Primers used in this study. (XLS 10 KB)

Below are the links to the authors’ original submitted files for images.

Authors’ original file for figure 1

Authors’ original file for figure 2

Authors’ original file for figure 3

Authors’ original file for figure 4

## Abbreviations

3-AT: 3-aminotriazole
AD: Activation domain
BD: Binding domain
CMS: Cytoplasmic male sterility
RF: Restorer of fertility
X-α-Gal: 5-Bromo-4-chloro-3-indolyl-α-D-galactopyranoside
Y2H: Yeast two hybrid
