# Supplementary figures and images for: A candidate factor that interacts with RF2, a restorer of fertility of Lead rice-type cytoplasmic male sterility in rice
Source: Rice (N Y). 2014 Oct 7;7:21. doi: 10.1186/s12284-014-0021-6 (PMC4884035; doi:10.1186/s12284-014-0021-6)

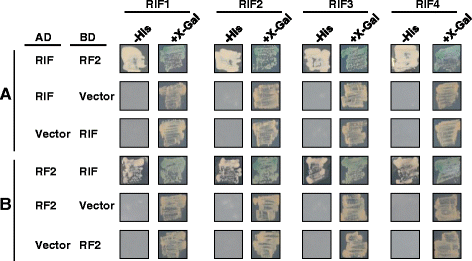

Supplement: Supplementary file 2 — Authors’ original file for figure 1 [file 12284_2014_21_MOESM2_ESM.gif]

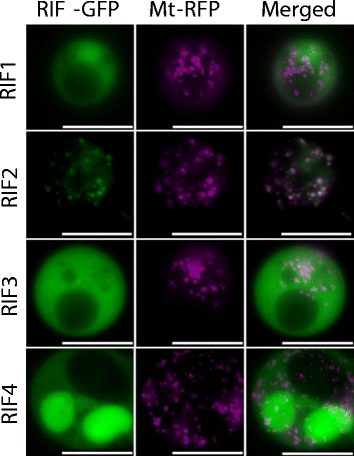

Supplement: Supplementary file 3 — Authors’ original file for figure 2 [file 12284_2014_21_MOESM3_ESM.gif]

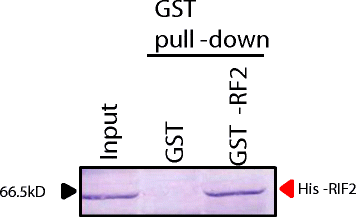

Supplement: Supplementary file 4 — Authors’ original file for figure 3 [file 12284_2014_21_MOESM4_ESM.gif]

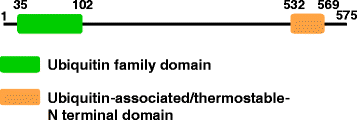

Supplement: Supplementary file 5 — Authors’ original file for figure 4 [file 12284_2014_21_MOESM5_ESM.gif]
